# Editorial: Recent Advances in Pheochromocytoma and Paraganglioma: Molecular Pathogenesis, Clinical Impacts, and Therapeutic Perspective

**DOI:** 10.3389/fendo.2021.720983

**Published:** 2021-08-23

**Authors:** Farhadul Islam, Ichiro Abe, Suja Pillai, Robert A. Smith, Alfred King-Yin Lam

**Affiliations:** ^1^Department of Biochemistry and Molecular Biology, University of Rajshahi, Rajshahi, Bangladesh; ^2^Department of Endocrinology and Diabetes Mellitus, Fukuoka University Chikushi Hospital, Chikushino, Japan; ^3^School of Biomedical Sciences, Faculty of Medicine, University of Queensland, Herston, QLD, Australia; ^4^Genomics Research Centre, Centre for Genomics and Personalised Health, Queensland University of Technology, Brisbane, QLD, Australia; ^5^Cancer Molecular Pathology of School of Medicine and Dentistry, Griffith University, Gold Coast, QLD, Australia

**Keywords:** pheochromocytoma, paraganglioma, neuroendocrine tumors, cancer therapeutics, cancer management

In this Research Topic, we have collected recent developments in research into Pheochromocytomas and Paragangliomas (PPGLs), highlighting their molecular mechanisms, clinical manifestations, and improved therapeutic management.

PPGLs are the primary types of neuroendocrine tumors, and are relatively rare, originating from chromaffin tissue in the adrenal medulla and/or autonomic nervous system ganglia (1). The symptoms experienced by PPGL patients include, but are not limited to, secretion of excess catecholamines, which can manifest as various cardiovascular-related indications such as hypertension *via* increased total peripheral resistance, heart attack without previous history, shock (non-cardiogenic pulmonary), arrhythmias, and oedemas (2). Also, pseudo-bowel obstructions, diabetic ketoacidosis, and multisystem crises involving lactic acidosis have been associated with PPGL patients (3). Since PPGLs are rare and symptoms are non-specific, they have potential for underdiagnosis. This may lead to the progression of undetected tumors into highly metastatic phenotypes in patients with PPGLs. Recent advances in understanding of the molecular biology of PPGLs, however, offer potential to open pathways to improve therapeutic interventions for PPGL tumors (4–9).

In this Research Topic, Ma et al. reported the genetic profiling of Chinese PPGL patients (n=314) examined by next-generation and Sanger sequencing. They noted that 29% of patients with PPGLs had shown mutations in one of several pathogenic genes. Among the tested genes, *SDHB* was the most frequently (14.6%) mutated. In addition, Ma et al. observed that metastatic PPGLs, paragangliomas, and younger patients (under 30 years of age) were more likely to harbor pathogenic mutations of the tested genes. Furthermore, Yamazaki et al. summarized the clinical significance of genetic abnormalities and their association with the phenotype of patients with PPGLs. Moreover, Saddozai et al. identified two distinct subtypes of PPGLs based on the expression of genes in two cohorts (data retrieved from the TCGA and GSE19422 databases).

Surgery is the mainstay of treatment for patients with PPGLs. Without proper preoperative preparation, the excessive release of catecholamines during tumor surgery can result in lethal cardiovascular complications (Fang et al.). Thus, Fang et al. reviewed the recent advancements in preoperative management including hypertension control and improvement of blood volume for patients with PPGLs. They summarize the available approaches and evidence for preoperative management of PPGLs with or without α adrenergic-receptor antagonists, which could facilitate improvements in outcomes for patients with PPGLs. Another study in this Research Topic reported on effective preoperative management approaches, in particular red blood cell transfusion during surgery on patients with PPGLs (Guo et al.). Therefore, the new methodologies detailed in the papers in this Research Topic could facilitate the understanding of physicians for better management of patients with PPGLs. In addition, Abe et al. reviewed the current development of glucose intolerance in PPGLs for this Research Topic, and summarized its clinical significance in patients with PPGLs.

Pseudohypoxia plays important roles in the tumourigenesis of PPGLs (6). Mutations in Von Hippel-Lindau (VHL) and hypoxia-induced factor (HIF) related genes, including *PHD*, *VHL*, *HIF-2A* (*EPAS1*), and *SDHx*, which are known as VHL/HIF axis genes, are common in PPGLs (10). Also, VHL/HIF-mediated pseudohypoxia has a critical role in the pathogenesis of PPGLs (10). Peng et al. reviewed the recent studies highlighting the VHL/HIF axis and its genetic alterations in PPGLs. In addition, they discussed the underlying mechanisms of VHL/HIF axis-driven PPGL pathogenesis and summarized the therapeutics targeting this axis in cancer (Peng et al.). Furthermore, Gao et al. reported that the suppression of VHL induced increased cell proliferation and migration of pheochromocytoma cell lines by modulating genes associated with cell proliferation.

An effective histological risk stratification for prediction of clinical outcomes for patients with PPGLs has not yet been established. However, Yamazaki et al. summarized the proposed histopathological and clinicopathological scoring systems in the present Research Topic. In their work, they listed the limitations of each scoring method, such as their ability to predict the metastatic potential of tumors (Yamazaki et al.). Another study in this series examined the relationship between the tumor microenvironment in the form of immune and sustentacular cell makeup, as well as the angiogenic potential of PPGLs with histopathology using the scoring methods. They identified several relationships, such as an association between intratumoral hemorrhage and its pathological malignancy scores (Gao et al.).

Succinate plays critical roles in PPGL pathogenesis and the accumulation of succinate receptor 1 (SUCNR1) in *SDHx* mutated PPGLs has been previously reported (11). In this Research Topic, Matlac et al. reported the oncometabolic function of succinate in *SDHx* mutation driven PPGLs *via* SUCNR1 stimulation of the ERK pathway, and the capacity of SUCNR1 inhibitors to abrogate this effect.

Another paper in this Research Topic reported a case study of a patient undergoing systemic therapies for metastatic PPGL and reviewed the current development of novel agents under clinical trials, including therapeutics for metastatic PPGLs (Economides et al.).

In conclusion, the information presented in this Research Topic provides an underlying molecular and genetic spectrum of PPGLs, unveiling their clinical implications, thereby enriching our understanding of the pathogenesis of the disease, which could improve clinical outcomes in patients with PPGLs.

## Author Contributions

FI, IA, SP, RS, and AL conceptualized, designed, wrote, and approved the editorial. All the authors contributed to the manuscript and approved the submitted version.

## Conflict of Interest

The authors declare that the research was conducted in the absence of any commercial or financial relationships that could be construed as a potential conflict of interest.

## Publisher’s Note

All claims expressed in this article are solely those of the authors and do not necessarily represent those of their affiliated organizations, or those of the publisher, the editors and the reviewers. Any product that may be evaluated in this article, or claim that may be made by its manufacturer, is not guaranteed or endorsed by the publisher.
